# Nonspecific orbital inflammation and thyroid eye disease, a rare comorbidity: report of two cases and review of literature

**DOI:** 10.1186/s12886-021-02008-z

**Published:** 2021-06-05

**Authors:** Bahram Eshraghi, Amin Dehghan, Niloofar Javadi, Mohammadreza Fazel

**Affiliations:** 1 Isfahan Eye Research Center, Department of Ophthalmology, Isfahan University of Medical Sciences, Isfahan, Iran; 2 Student Research Committee, School of Medicine, Isfahan University of Medical Sciences, Isfahan, Iran

**Keywords:** Non-specific orbital inflammation, thyroid eye disease, comorbidity, orbital mass, prednisolone

## Abstract

**Background:**

To present the very rare comorbidity of developing non-specific orbital inflammation (NSOI) in two patients with histories of definite thyroid eye disease (TED).

**Case presentation:**

Both patients complained of new-onset progressive proptosis although their thyroid disease was controlled and computed tomography scan revealed an intraorbital inflammatory mass. The pathological assessment indicated that both patients had developed fibrosing NSOI. Therefore, intravenous corticosteroids were administered. The mass regressed and the amount of proptosis was decreased in both patients.

**Conclusions:**

We reviewed all related cases in the literature and extracted their clinical and radiological characteristics for this paper. Ophthalmologists should consider TED and NSOI in patients with a new-onset complaint of proptosis. Despite rare comorbidity of TED and NSOI, it should be considered especially in patients with refractory proptosis, and lead to its further evaluation and prompt management.

## Background

Thyroid eye disease (TED) is an autoimmune orbital inflammatory disorder associated with thyroid inflammatory diseases. Sight-threatening TED occurs in 3-7 % of cases due to optic nerve compression or corneal exposure, but the majority of the cases are mild and self-limiting. The most common ocular sign of TED is upper eyelid retraction, occurring in 90 % of the patients, followed by proptosis and ocular motility restriction. Shan et al. reported that deposition of hyaluronan (a hydrophilic glycosaminoglycan) between extraocular muscle fibers as the main histopathologic findings in TED, which may cause proptosis and ocular motility restriction. Besides, infiltration of inflammatory cells and their cytokines are responsible for soft tissue enlargement. Since the orbital walls consist of several rigid bones, the events mentioned causing increased intraorbital pressure [1]. Orbital imaging, particularly computed tomography scan (CT-Scan) and magnetic resonance imaging (MRI), may be necessary to confirm the diagnosis. Treatment strategy includes medical interventions to normalize the thyroid function tests and suppress orbital inflammation in the active phase of the disease. Surgical procedures are performed only under specific conditions [2, 3].

Idiopathic orbital inflammatory disease, also known as non-specific orbital inflammation (NSOI), is a benign, non-infectious inflammatory orbital disorder that is not associated with any systemic or local etiologies. This disease accounts for about 8 % to10 % of orbital masses, proving it to be one of the most common causes of proptosis in adults. NSOI may have various presentations, such as inflammation of the lacrimal gland in its most common form- known as dacryoadenitis, myositis of one or more extraocular muscles (EOMs), and some other rare presentations [4]. There are rare case reports of concomitant occurrence of an NSOI presentation with TED [5–10].

In the present study, we report two cases presenting with simultaneous NSOI and TED in their clinical courses. In addition, we review previous investigations that reported comorbidity of TED and any form of NSOI.

## Case presentation

### Case 1

A 50-year-old man with a history of hyperthyroidism and smoking presented to our clinic with bilateral proptosis for the past year. He received a single-dose iodine therapy after Graves’ disease was diagnosed for him 10 years ago, and he had been on anti-thyroid medication (methimazole) ever since. He was otherwise healthy.

The best corrected visual acuity (BCVA) was 20/20, and the relative afferent pupillary defect (RAPD) was 1 + positive in the left eye (OS). He had developed 2 mm superior eyelid retraction OS, with a clinical activity score (CAS) of 0 out of 7 on both sides. The degree of proptosis measured by the Hertel exophthalmometer was 26 mm and 27 mm in his right eye (OD) and OS, respectively. Ocular motility was mildly restricted in superior gazes on both sides. Anterior segment examination, color vision, intraocular pressure (IOP) and dilated fundus examination were all within normal limits in both eyes. A generalized depression visual field defect was detected in his left side.

**Fig. 1:**
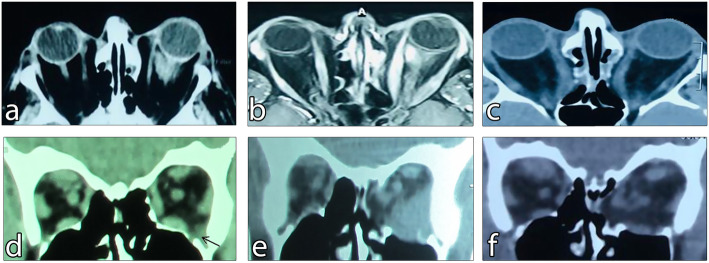
**A** Baseline CT-scan, revealed an infiltrative mass with intraconal and extraconal expansion in the 1st patient. **B** Baseline T1- weighted with fat suppression MRI with gadolinium-based contrast agents showed a hyper-intense infiltrative lesion in the left orbit in addition to EOM enlargement in the 1st patient. **C** Mass resolution was determined in the CT-Scan performed four years after intravenous corticosteroid injection followed by orbital decompression in the 1st patient. **D** Baseline CT-scan revealed a very small infratemporal mass as indicated by the arrow which remained undiagnosed until its progression in the 2nd patient. **E** six months’ post decompression surgery CT-scan, showed infiltrative mass enlargement in the 2nd patient. **F** The lesion was resolved after intravenous corticosteroid administration in the 2nd patient.

CT-Scan revealed an intraorbital infiltrative mass that expanded to intraconal and extraconal spaces with no EOM enlargement (Fig. 1A). MRI with gadolinium-based contrast agents revealed a hyper-intense infiltrative lesion in T1- weighted with fat suppression images (Fig. 1B). Based on the clinical findings, the patient underwent surgical procedures for medial and inferior wall orbital decompression OD and biopsy of the orbital mass OS in one session. Pathological assessments confirmed the diagnosis of simultaneous TED and chronic fibrosing NSOI, so 1 gr intravenous methylprednisolone per day was administered for three consecutive days.

Further rheumatologic investigations revealed normal antibody profile. Examination of the patient five-month post-operative revealed resolution of the orbital mass as well as a normal visual field.

One month later, the patient underwent decompression surgery in the inferior wall OD and medial wall OS to manage the remained proptosis. After the second surgery, the patient was visited at regular intervals for four years, and Hertel exophthalmometer revealed proptosis of 21.5 mm in both eyes with no ocular motility restriction bilaterally. No mass was observed in CT-scan (Fig. 1C).

### Case 2

A 23-year-old woman was referred to the oculoplastic clinic of Feiz Eye Hospital, Isfahan, Iran with the chief complaint of gradual progressive proptosis that had started in her left eye a year before the visit. Despite being euthyroid, the anti-TPO antibody was positive in the patient’s laboratory tests and she had a history of thyroid inflammation during the previous four years. She did not take any anti-thyroid medications, and the smoking history was negative.

BCVA was 20/20 and RAPD was negative bilaterally. Furthermore, she had 1 mm superior eyelid retraction OD and 3 mm superior eyelid retraction OS, and CAS of 0 out of 7 bilaterally. The degree of proptosis measured by the Hertel exophthalmometer was 20 mm OD and 22 mm OS. Anterior and posterior segments did not have remarkable findings and IOP was normal in both eyes. CT-scan findings were also unremarkable. Based on the clinical findings, we decided to perform a medial wall decompression OS to resolve the proptosis.

The amount of proptosis was not significantly reduced during the 6-month postoperative visits, and severe ocular motility restriction and pain were also detected in all superior directions OS. The previous CT-scan was re-examined, and a small infratemporal infiltrative mass was detected (Fig. 1D). A new CT-scan was performed to evaluate the condition and it revealed the enlargement of the previously undiagnosed lesion (Fig. 1E). Based on the clinical and radiologic findings, the diagnosis of NSOI associated with TED was highly suspected, and the patient underwent orbital biopsy. The pathological assessment revealed that the orbital mass was a fibrous NSOI, confirming our clinical diagnosis, and further rheumatologic assessments were normal. To suppress the inflammation, 500 mg intravenous methylprednisolone was administered for 3 consecutive days and the mass was completely resolved (Fig. 1F). The patient was followed for two years when she developed a mass in the left sub-brow area extending to the forehead. The pathology report of the surgical biopsy sample confirmed chronic NSOI. Oral methylprednisolone 1 mg/kg was administered and gradually tapered over three months. In addition, mycophenolate mofetil 500 mg every 12 h was also administered for four months. The proptosis was not increased and her clinical condition remained stable.

## Discussion

Described for the first time in 1905, NSOI refers to orbital inflammation without any specific local or systemic underlying pathologies. It is diagnosed if other similar conditions are ruled out including TED, orbital lymphoproliferative diseases, and other systemic inflammations involving the orbit [11, 12].

Both of the cases presented in this report had a definite diagnosis of TED, and NSOI was subsequently diagnosed for them following new clinical manifestations. TED and NSOI can occur in the same patient at different times. For example, Kubota et al. reported a patient with a history of Grave’s ophthalmopathy presenting with an upper eyelid mass, for which pathological and serological assessments revealed the diagnosis of IgG4-related ocular disorder [7].

To date, only six patients with simultaneous NSOI and TED have been reported in the literature [7–10]. This comorbidity can occur in any thyroid function status, for example, hyperthyroidism as seen in our first patient. The second reported case here was euthyroid with positive anti-TPO antibodies before the new-onset left proptosis. Hyperthyroidism, hypothyroidism, and euthyroidism have been observed in six [5, 7, 8, 10], two [5], and one [5] previously reported patients, respectively. However, the thyroid status was not determined in patients reported by Detorakis et al. and Shieh et al. [6, 9].

According to review of the literature, comorbidity of NSOI and TED occurs almost equally among men and women with a ratio of 6:5. The median age of the previously reported patients was 47, ranging between 22 and 68 years [5–10]. Both our cases presented with the chief complaint of a new-onset proptosis. As described in Table 1, proptosis was reported in all but two of the previous patients [7, 10]. For example, Tachibana et al. described a patient presenting with severe ocular motility restriction and pain without proptosis. Their radiologic assessment revealed the superior rectus muscle enlargement, which led to the diagnosis of TED associated with myositis [7].

**Table 1:** Demographic, clinical and laboratory data and outcomes of previous reported cases with simultaneous NSOI and TED

Year	First Author	Number	Age	Sex	History	Thyroid status	1st CC[1]	Examinations	Radiologic findings	Diagnosis	Treatment	Outcome	Interval	2nd CC	Examinations	Radiological findings	Diagnosis	Treatment	Outcome	Interval	3rd CC	Examinations	Radiological findings	Diagnosis	Treatment	Outcome
2010	Bijlsma WR.	1	47	F[2]	NR[3]	Autoimmune hypothyroidism	Progressive painless proptosis (OS)	NR	A posterior superior orbital mass (OS)	NSOI[4]	Oral prednisone	Complete resolution	11 months	NR	NR	Diffuse retro bulbar mass	NSOI (OD)	Oral prednisone	NR	4 years	Eyelid retraction + proptosis (OD)	NR	EOM[5]enlargement (OU)	GO[6] (Positive ATA[7])	Surgical correction of eyelid retraction	Complete resolution
		2	35	F	NR	Primary hypothyroidism	Subacute eyelid swelling + proptosis + eyeball motility restriction + pain (OD)	NR	lacrimal gland enlargement (OD)	NSOI (dacryoadenitis)	Methyl prednisolone IV	NR	4 months	Diplopia + Upper eyelid retraction (OS)	NR	EOM enlargement (OS)	Unilateral GO (Positive ATA)	Radiotherapy	NR	4 years	NR	NR	NR	NSOI (Dacryoadenitis)	Methyl prednisolone IV	NR
		3	30	M[8]	Diabetes mellitus + Crohn disease	Hyperthyroidism	Painless proptosis + eyeball motility restriction + upper eyelid retraction (OU)	NR	EOM enlargement (OU)	GO (Positive ATA)	-	Complete resolution without therapy	9 years	Proptosis + eyeball motility restriction (OS)	NR	Lacrimal gland enlargement (OS)	NSOI (Dacryoadenitis)	Oral prednisone	NR	2 months	Proptosis + eyeball motility restriction (OD)	NR	Lacrimal gland enlargement (OD)	NSOI (Dacryoadenitis)	Oral prednisone	NR
		4	22	M	NR	Euthyroidism	Proptosis + eyeball motility restriction + pain (OS)	NR	Medial superior orbital mass (OS)	NSOI (Fibrosing)	Radiotherapy + Oral prednisone	NR	5 years	Progressive proptosis (OS)	NR	EOM enlargement (OD)	GO (Positive ATA) (Normal TFT)	Surgical decompression	Complete resolution	-	-	-	-	-	-	-
2011	Kubota T.	5	64	F	NR	GO associated with hyperthyroidism (Positive ATA)	Upper eyelid mass (OS)	NR	Anterior superior orbital infiltrative lesions (OU)	NSOI (Ocular adnexal IgG4-related Inflammation)	Oral prednisone 30 mg/day with slow tapering	Partial resolution	-	-	-	-	-	-	-	-	-	-	-	-	-	-
2012	Tachibana S.	6	52	M	Hyperuricemia Partial hepatectomy Smoker	Hyperthyroid - Under treatment with Methimazol - Subclinical hypothyroidism at the time of complaint	Eye pain + Severe motility restriction (OS) + Diplopia	VA[9]: 20/20 (OD) 20/40 (OS) CAS[10]: 6/7 (OS) Ptosis + Lid retraction (OS) Exophthalmometer: 17 mm (OD) + 17 mm (OS)	SR[11]enlargement with tendon involvement	Simultaneous TED 12 and NSOI	Methyl prednisolone IV 1 gr 3 times/ week	Dramatic improvement after 2 months: VA: 20/25 (OD) 20/22 (OS) CAS: 0/7 (OS) Exophthalmometer: 17 mm (OD) + 17 mm (OS)	-	-	-	-	-	-	-	-	-	-	-	-	-	-
2014	Detorakis E.	7	46	M	Testicular seminoma surgically removed 20 years ago	NR	Proptosis (OU) + Ocular hypertension (OD)	VA; 20/25 (OD) 20/20 (OS) IOP:32 mmHg (OD) 17 mmHg (OS). Exophthalmometer: 28 mm (OD) + 26 mm (OS)	Fusiform EOM enlargement Specially in IR[13](OD) + Infratemporal intraorbital tumor in apposition with the posterior aspect of the globe (OD)	Simultaneous TED and Sclerosing NSOI	Oral prednisone 1 mg/kg (6 weeks)	Reduction in size and vascularity after 6 months IOP: 17 (OD)	-	-	-	-	-	-	-	-	-	-	-	-	-	-
2015	Shen T.	8	42	M	NR	Hyperthyroidism (5 years)	Proptosis for 2 years + Moderate upper eyelid retraction + Moderate motility restriction (OD)	VA: 25/30 (OD) 25/20 (OS) IOP: normal (OU) Exophthalmometer : 24 mm (OD) + 17 mm (OS)	MR [15] + LR[16]enlargement with tendon sparing (OD) + Ambiguous soft tissue mass of 1.8 × 1.5 cm in retro bulbar cone region (OD).	Simultaneous TED and NSOI	Methyl prednisolone IV 500 mg (5 days) + oral prednisone 50 mg (5 days) + tapering during 2 months	Complete resolution No recurrence after 6 months	-	-	-	-	-	-	-	-	-	-	-	-	-	-
		9	49	M	NR	Hyperthyroidism (8 years) - Under treatment with PTU	Proptosis for 8 years (OU) + Eye redness for 2 months (OU) + Mild diplopia for 2 months	VA: Normal (OU) Severe motility restriction (OU) Exophthalmometer: 27 mm (OD) + 28 mm (OS)	SR and IR enlargement with tendon sparing (OU) + Inferior orbital soft tissue mass of 2.1 × 1.4 cm (OD) + Inferior orbital soft tissue mass of 1.5 × 1.2 cm (OS)	Simultaneous TED and NSOI	Methyl prednisolone IV 500 mg (5 days) + oral prednisone 50 mg (5 days) + tapering during 1 months	Complete resolution	1 year	NR	NR	NR	IOID	Oral prednisone	NR	-	-	-	-	-	-	-
		10	48	F	NR	Hyperthyroidism (3 years)	Proptosis (OU)	VA: Normal (OU) Orbital masses in lacrimal area (OU) + Inferior orbital mass (OS) Mild motility restriction in medial & lateral gaze (OS) + lateral gaze (OD) Exophthalmometer: 17 mm (OD) + 20 mm (OS)	LR & MR enlargement with tendon sparing (OS) Inferior orbital soft tissue mass of 1.5 × 1.2 cm (OS) MR enlargement (OD) 2 well-defined homogeneous soft tissue masses of 1.1 × 1.0 cm and 0.9 × 0.6 cm in lacrimal gland areas (OU)	Simultaneous TED and NSOI	Dexamethasone IV 10 mg (5days) + oral prednisone 40 mg (5 days)	Complete resolution	-	-	-	-	-	-	-	-	-	-	-	-	-	-
2017	Shieh WS.	11	68	F	Well controlled hypertension Smoker	NR	Progressive proptosis (OD)	VA: 20/40 (OD) 20/25 (OS0 Inferior & lateral gaze restriction (OD) Upper eyelid retraction + lagophthalmos (OD)	SR & IR & MR enlargement with tendon sparing (OD) well circumscribed superior orbital mass (OD) lacrimal gland enlargement (OD) Maxillary sinus opacification & orbital floor bowing & bony erosion (OU)	Simultaneous TED and NSOI (dacryoadenitis) + Silent sinus syndrome	Endoscopic sinus drainage surgery & endoscopic medial orbital decompression (OD) & Oral prednisone	Partial resolution of proptosis	-	-	-	-	-	-	-	-	-	-	-	-	-	-

1- Chief complaint 2- Female 3- Not reported in the article 4- Nonspecific orbital inflammation 5- Extraocular muscles 6- Grave’s ophthalmopathy 7- Anti-thyroid antibodies 8- Male 9- Visual acuity 10- Clinical activity score 11- Superior rectus muscle 12- Thyroid eye disease 13- Inferior rectus muscle 14- Medial rectus muscle 15- Lateral rectus muscle

Patients with orbital involvement and a history of any thyroid inflammation are highly suspected of TED. In addition, certain clinical manifestations and specific radiological findings may help differentiate TED from other orbital lesions [2, 3]. Upper eyelid retraction, which is the most frequent sign in patients with TED [2], was detected in both our cases. This finding is not usually observed in NSOI. When extraocular muscles are involved, tendons are usually spared in TED but not in NSOI [2, 11, 12]. The presence of orbital masses are highly associated with NSOI or other orbital malignancies but not with TED [4, 11–13].

Shen et al. reported three hyperthyroid patients with CT-scan reports of extraocular muscle enlargement, tendon sparing and intraorbital inflammatory masses, which resulted in the diagnosis of simultaneous TED and NSOI [9].

Corticosteroids are the first-line treatment for the active phase of NSOI and TED [2, 4]. Except for the sclerosing form, other types of NSOI dramatically respond to intravenous or oral steroids, and some researchers believe that the response rate to corticosteroids is a promising way to differentiate between TED and NSOI [11, 12]. The molecular mechanism of action in corticosteroid therapy in these patients is not completely known yet. Future molecular studies should address how corticosteroids therapy resolves orbital inflammatory masses, possibly through RNA-Seq experiments, like the studies on the genes involved in retinal dystrophies and retinitis pigmentosa [14–16]. As in the previous reports, we tried corticosteroids as the first line treatment to manage our patients. The diagnosis of fibrosing NSOI associated with TED was confirmed in both our patients. As expected, the orbital inflammatory masses were resolved in both cases.

New clinical manifestations in patients with controlled thyroid status and TED suggests the need for new imaging and even surgical biopsies if a mass is reported. Rheumatologic assessments appear to be mandatory in those with NSOI in the histopathological reports. Finally, TED and NSOI should be treated as independent diseases, however their pathophysiology and treatments may overlap.

## Conclusions

Comorbidity of NSOI and TED, appearing either at the same time or at different periods, is really rare and occurs through unknown mechanisms. Ophthalmologists should be aware of this comorbidity and its influence on patient’s responses to conventional treatments. This comorbidity should be considered in poorly responsive or unresponsive patients. The correct diagnosis requires detailed history, accurate physical examination and appropriate diagnostic evaluations in such patients. Furthermore, more extensive studies with specific histopathological and molecular evaluations should be conducted to determine the mechanisms leading to NSOI and TED and to discover whether their simultaneous occurrence reflects underlying connections or is merely coincidental.

## Data Availability

All data generated or analyzed during this study are included in this published article.

## Abbreviations

TED: Thyroid Eye Disease
CT-Scan: Computed Tomography Scan
MRI: Magnetic Resonance Imaging
NSOI: Non-Specific Orbital Inflammation
EOM: Extraocular Muscles
BCVA: Best Corrected Visual Acuity
RAPD: Relative Afferent Pupillary Defect
CAS: Clinical Activity Score
IOP: Intraocular Pressure
Anti-TPO: Anti-Thyroid Peroxidase
